# Supplementary material for: Increasing the biocompatibility of graphene-based hybrid nanostructures with glycopolymer
Source: Turk J Chem. 2021 Nov 29;46(2):404–14. doi: 10.3906/kim-2109-60 (PMC10734709; doi:10.3906/kim-2109-60)
Supplement: Supplementary file 1 [file TJC2204_SP-KIM-2109-60_manuscript_rev-s.docx]

Supporting Information

Increasing the Biocompatibility of Graphene Based Hybrid Nanostructures with Glycopolymer

Aydan DAG^1,2^*, Pınar Sinem OMURTAG ÖZGEN^3^, Belma ZENGIN KURT^1^, Zehra DURMUS^4,5^

^1^ Department of Pharmaceutical Chemistry, Faculty of Pharmacy, Bezmialem Vakif University, Istanbul, Turkey

^2^ Drug Application and Research Center, Bezmialem Vakif University, Istanbul, Turkey

^3^ Department of Analytical Chemistry, School of Pharmacy, Istanbul Medipol University, Istanbul, Turkey

^4^ Baglar Mah., Gunesli Konutlar, No: 38, D-24, Istanbul, Turkey

^5^ Centre for Innovation Competence (ZIK) SiLi‐nano, Martin Luther University Halle‐Wittenberg, Halle (Salle), Germany

*Correspondence: [adag@bezmialem.edu.tr](mailto:adag@bezmialem.edu.tr)

| **A)** |  |
| --- | --- |
| **B)** |  |

**Scheme S1.** Schematic representation of synthesis of glycoblock copolymers

1. **Materials**

Sulfuric acid, hydrazine hydrate, potassium permanganate, sodium nitrate, sodium hydroxide, hydrochloric acid (37%), hydrogen peroxide (30%) and ammonia solutions (28%) were purchased from Sigma Aldrich. A commercial-grade, thermally expanded graphite (EG) powder, TIMREX® BNB90, kindly provided by IMERYS Carbon & Graphite (Switzerland). Unless otherwise indicated, all the reagents and solvents were purchased from Sigma-Aldrich or Merck and used as received.

1. **Characterization Techniques and Characterization**

**Fourier Transform Infrared (FTIR) Spectroscopy:** FTIR spectra were recorded on Bruker Alpha infrared spectrometer equipped with an attenuated total reflectance (ATR) device and germanium crystal was used. FTIR spectra of samples were recorded within a wave number range of 500-4000 cm^-1^ with a resolution of 4 cm^-1^ from 16 scans in transmission mode. The spectra were analyzed by using the “OPUS” and Origin v8.5 software.

**Nuclear Magnetic Resonance (NMR) Spectrometry:** NMR general characterization was conducted using a Bruker BioSpin AG Avance 500 mHz Spectrometer (^1^H (500 MHz), ^13^C (125 MHz)). Samples were analyzed in the solvents of CDCl_3_ and DMSO-d_6_. All chemical shifts are stated in ppm (δ) relative to Si(CH_3_)_4_ as internal standard (δ = 0.00 ppm), referenced to the chemical shifts of residual solvent resonances (^1^H and ^13^C).

**Gel permeation chromatography (GPC):** The molecular weight and polydispersity of synthesized polymers were analyzed via gel permeation chromatography (GPC). A Viscotek GPCmax modular system comprising a VE 2001 autoinjector, Viscotek VE 3580 refractive index (RI) detector was used. A Viscotek CLM3008 guard column (4.6 × 10 mm) followed by three 300 × 7.8 mm linear columns (T3000, LT4000L, and LT5000L), (7.8x 300 mm) were employed for analysis. Tetrahydrofuran (THF, HPLC grade, 0.05% w/v 2,6-dibutyl-4-methylphenol (BHT)) with a flow rate of 1 mL/min at 35 °C was used as mobile phase. Fifty microliters of polymer solution sample with a concentration of 4-5 mg/ mL in THF was used for every injection. The calibration was performed using commercially available narrow-polydispersity polystyrene (PS) standards (Polymer Laboratories).

**Thermogravimetric analysis (TGA):** The thermal stability was determined by TGA with a Perkin Elmer Instruments model, STA 6000 for 5 mg of powder sample at a heating rate of 10 °C/min. in the temperature range of 30-800 °C under nitrogen atmosphere.

**Transmission Electron Microscopy (TEM):** Transmission electron microscope (TEM) micrographs were obtained using a JEOL JEM 2100 microscope. The instrument operates at an accelerating voltage of 200 kV. Samples were imaged without any staining.

**UV-Visible spectrophotometer**: UV-Vis spectra were recorded on a Hitachi U-2900 UV-Vis spectrophotometer.

**Ultrasonic Homogenizer:** Bandelin mark SONOPULS HD 2200 ultrasonic homogenizer was used with ultrasonic horn within the 50% amplitude which produces 20 kHz uniform sonic waves.


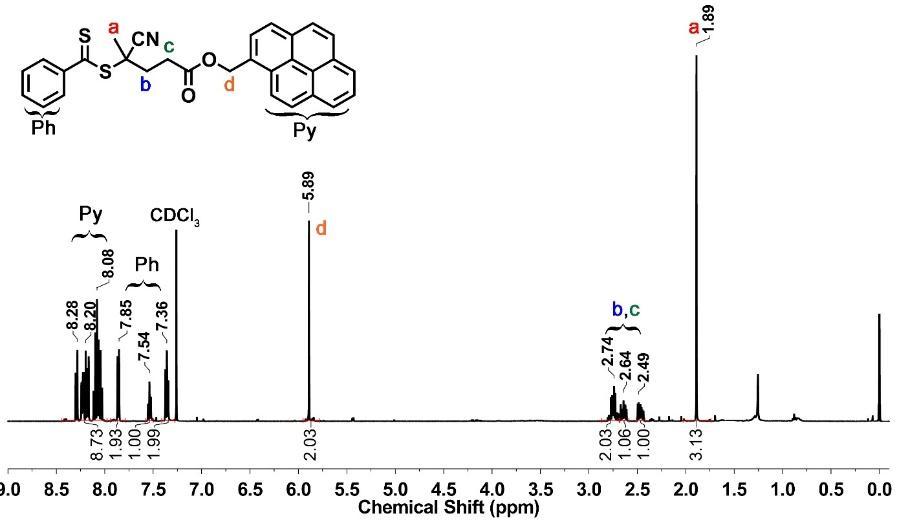


**Figure S1.** ^1^H-NMR spectrum of pyren-1-ylmethyl 4-cyano-4-((phenylcarbonothioyl)thio) pentanoate (CPADB-py) in CDCl_3_ (500 MHz).


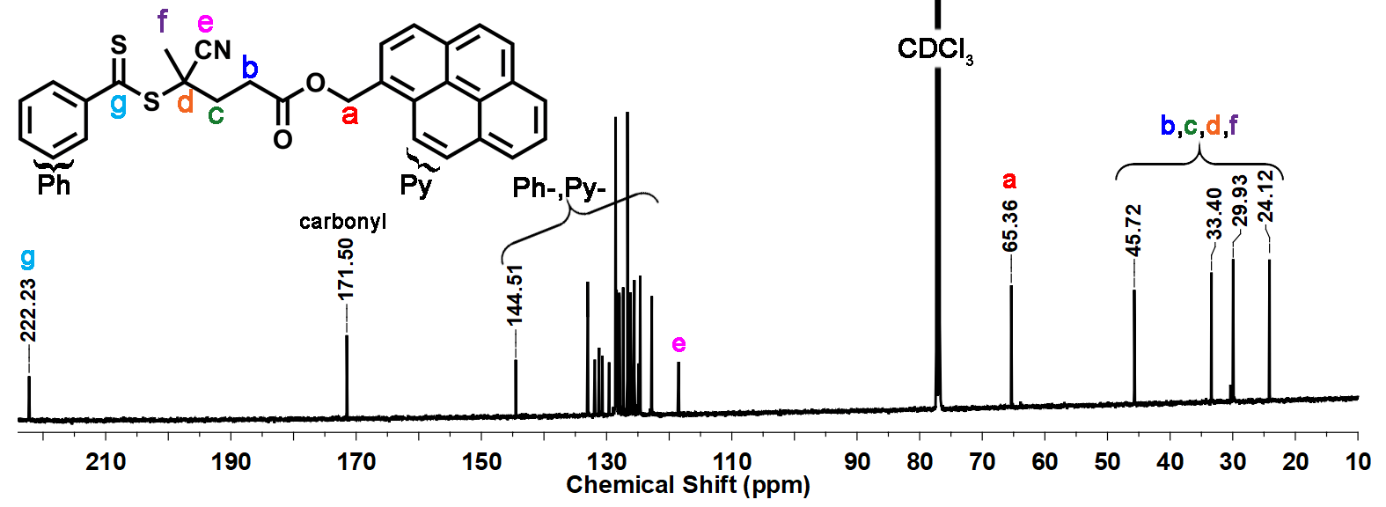


**Figure S2.** ^13^C-NMR spectrum of pyren-1-ylmethyl 4-cyano-4-((phenylcarbonothioyl)thio) pentanoate (CPADB-py) in CDCl_3_ (125 MHz).


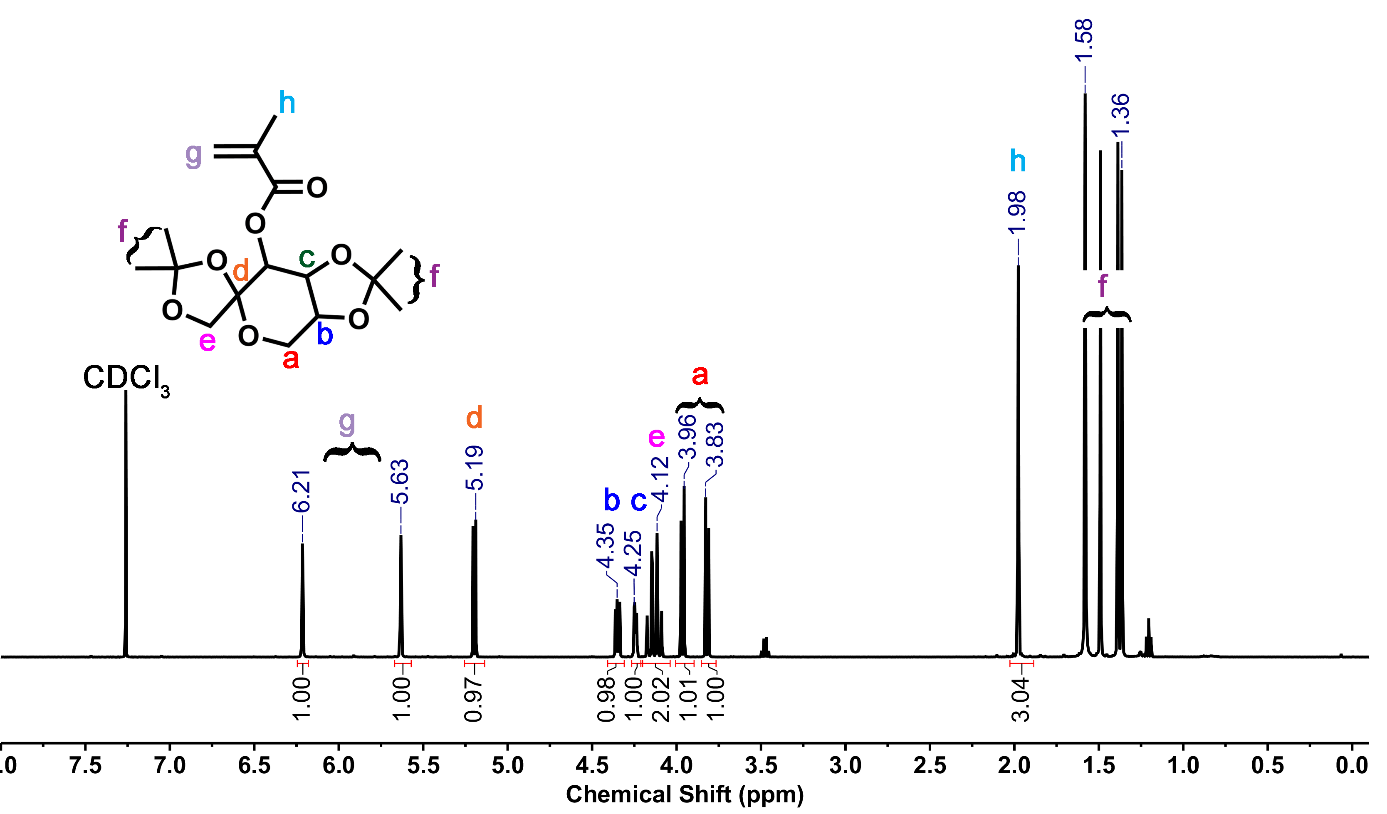


**Figure S3.** ^1^H-NMR spectrum of 3-*O*-methacryloyl 1,2:4,5-di-*O*-isopropylidene-β-D-fructopyranose (*ipr*Fruc_1,2_MA) in CDCl_3_ (500 MHz).


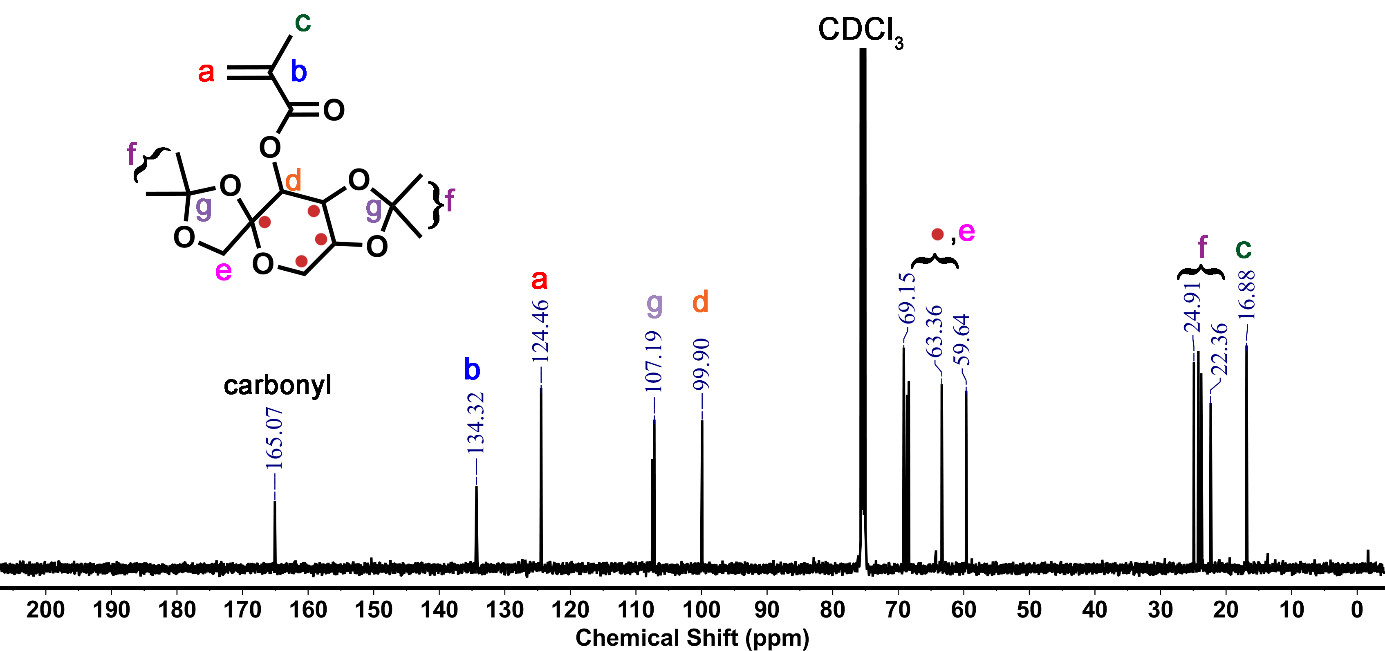


**Figure S4.** ^13^C-NMR spectrum of 3-*O*-methacryloyl 1,2:4,5-di-*O*-isopropylidene-β-D-fructopyranose (*ipr*Fruc_1,2_MA) in CDCl_3_ (125 MHz).


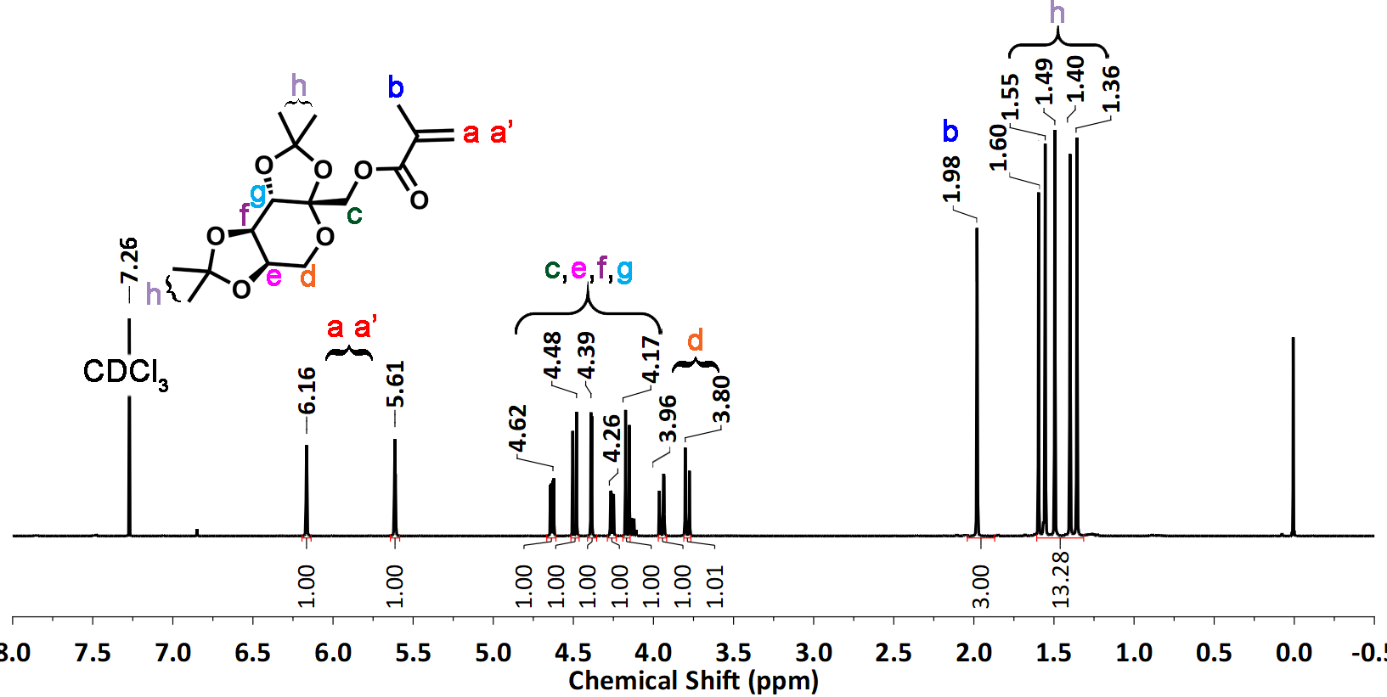


**Figure S5.** ^1^H-NMR spectrum of 1-*O*-methacryloyl 2,3:4,5-di-*O*-isopropylidene-β-D-fructopyranose (*ipr*Fruc_2,3_MA) in CDCl_3_ (500 MHz).


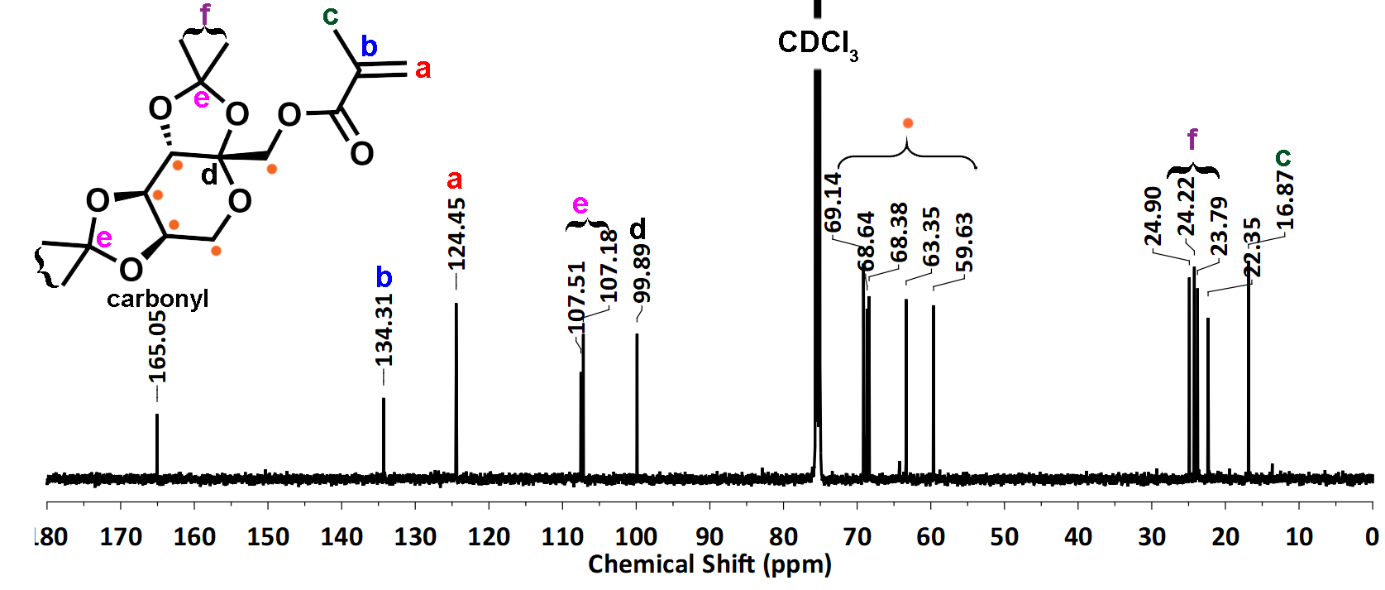


**Figure S6.** ^13^C-NMR spectrum of 1-*O*-methacryloyl 2,3:4,5-di-*O*-isopropylidene-β-D-fructopyranose (*ipr*Fruc_2,3_MA) in CDCl_3_ (125 MHz).

| **A)** | 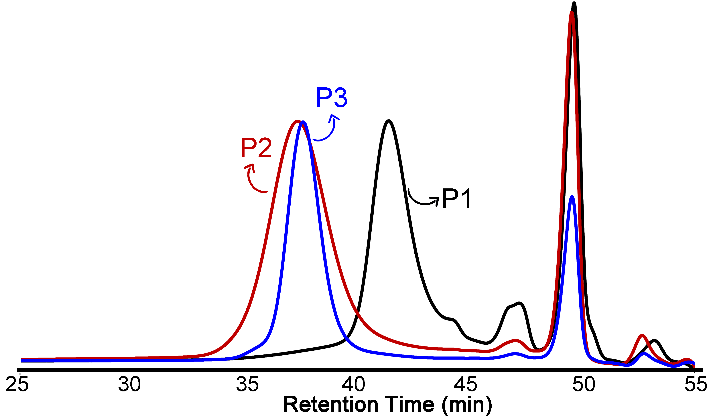 | **B)** | 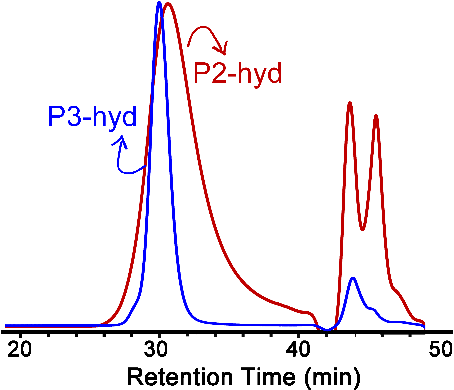 |
| --- | --- | --- | --- |

**Figure S7.** GPC chromatograms of **A)** P1, P2, P3 measured by THF-GPC and **B)** P2-hyd and P3-hyd measured by DMF-GPC.


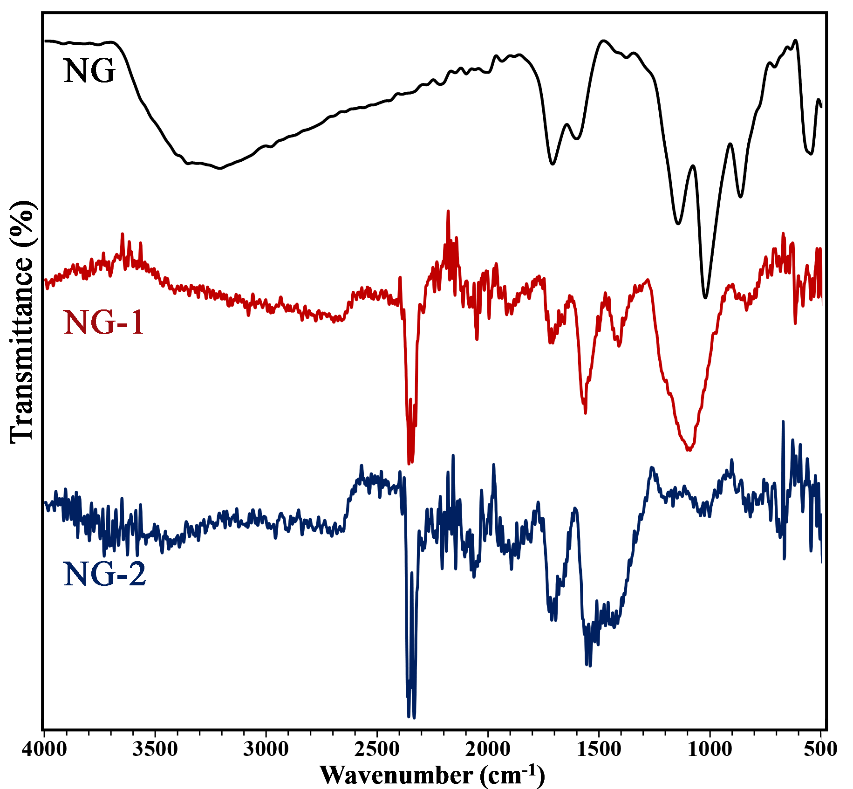


**Figure S8.** FT-IR spectra overlay of nanographene (NG) and glycoblock copolymer modified NGs.
